# The antigen recognition portion of African buffalo class I MHC is highly polymorphic, consistent with a complex pathogen challenge environment, and the 3’ region suggests distinct haplotype configurations

**DOI:** 10.1007/s00251-022-01287-0

**Published:** 2022-12-13

**Authors:** Isaiah Obara, Ard Nijhof, Patrick Atimnedi, Domnic Mijele, Anne Nanteza, Khawla Elati, Richard Bishop

**Affiliations:** 1 Freie Universität Berlin, Institute for Parasitology and Tropical Veterinary Medicine, Department of Veterinary Medicine, Berlin, Germany; 2 Freie Universität Berlin, Veterinary Centre for Resistance Research, Department of Veterinary Medicine, Berlin, Germany; 3 Department of Veterinary Microbiology and Pathology, Washington State University, Pullman, WA USA; 4 Uganda Wildlife Authority, Kampala, Uganda; 5 Kenya Wildlife Service, Nairobi, Kenya; 6 College of Veterinary Medicine, Animal Resources and Biosecurity, Makerere University, Kampala, Uganda; 7 Laboratoire de Parasitologie, Institution de La Recherche Et de L’Enseignement Supérieur Agricoles &, Univ. Manouba, École Nationale de Médecine Vétérinaire de Sidi Thabet, Sidi Thabet, Tunisia

**Keywords:** African buffalo (*Syncerus caffer*), Class I MHC, *Theileria parva*

## Abstract

African buffalo (*Syncerus caffer)* have been distinct from the Auroch lineage leading to domestic cattle for 5 million years, and are reservoirs of multiple pathogens, that affect introduced domestic cattle. To date, there has been no analysis of the class I MHC locus in African buffalo. We present the first data on African buffalo class I MHC, which demonstrates that gene and predicted protein coding sequences are approximately 86–87% similar to that of African domestic cattle in the peptide binding region. The study also shows concordance in the distribution of codons with elevated posterior probabilities of positive selection in the buffalo class I MHC and known antigen binding sites in cattle. Overall, the diversity in buffalo class I sequences appears greater than that in cattle, perhaps related to a more complex pathogen challenge environment in Africa. However, application of NetMHCpan suggested broad clustering of peptide binding specificities between buffalo and cattle. Furthermore, in the case of at least 20 alleles, critical peptide-binding residues appear to be conserved with those of cattle, including at secondary anchor residues. Alleles with six different length transmembrane regions were detected. This preliminary analysis suggests that like cattle, but unlike most other mammals, African buffalo appears to exhibit configuration (haplotype) variation in which the loci are expressed in distinct combinations.

## Introduction

Class I MHC molecules are membrane-bound surface glycoproteins, present on the great majority of nucleated cells, that comprise a key component of mammalian immune surveillance of infectious diseases and tumours. This is implemented through binding of self and foreign peptides derived from pathogens, enabling recognition by CD8^+^ T cells and induction of immune responses. They possess a structure consisting of a three-domain alpha heavy chain (α1, α2 and α3), encoded within the MHC region, non-covalently bound to the β2-microglobulin light chain (β2M). The hyper-variable amino-terminal α1 and α2 domains in the heavy chain of class I MHC molecules form a peptide-binding groove, that non-covalently binds peptides which are typically 8–10 (sometimes 11) amino acids in length.

The MHC multicopy gene family is highly polymorphic, both within and between species, enabling binding of a diverse array of both host-derived and foreign peptides for recognition by mammalian T cells. The class I MHC genes and predicted proteins of a variety of mammalian families including primates, rodents, suids, and bovids have been sequenced, and very high levels of allelic polymorphism identified. Within the family, Bovidae, the subfamily Bovinae, contains several distinct ‘tribes’ defined by morphology combined with sequencing of mitochondrial genomes and selected nuclear genes that diverged from one another at least 5 million years ago. Sequences have been determined for class I loci from several species of the Bovinae, including American bison (*Bison bonasus*, Babik et al. 2012) and domestic cattle (*Bos taurus* and *Bos indicus*, Ellis et al. 2004), which cluster together in a single tribe. The class I MHC genes of domestic cattle are unusual in that there are six loci which exhibit ‘haplotype diversity’, meaning that in addition to allelic polymorphism, the exact complement of expressed loci is variable between different animals. Class I MHC sequence diversity has not yet been analysed for the African buffalo (*Syncerus caffer*) which clusters together with Asian water buffalo (*Bubalus bubalis*) in a separate tribe within the Bovinae (Matthee and Davis 2001).

African buffalo are distributed from Sudan, Uganda, and Kenya to South Africa, and also in the savannah regions of West Africa. It is the major mammalian wildlife host of multiple species of livestock-infective apicomplexan protozoa in the genus *Theileria*, including *Theileria parva* and *Theileria mutans* in Eastern and Southern Africa (Norval et al. 1992; Young et al. 1978). Transmission of *T. parva* between domestic cattle by the tick *Rhipicephalus appendiculatus* typically presents as a fatal disease termed East Coast Fever (ECF). The disease is characterized by high schizont parasitosis in lymphocytes and high piroplasm parasitaemia in erythrocytes (Norval et al. 1992). Cattle exposed to ticks that have previously fed on *T. parva-*infected buffalo develop a distinct clinical syndrome, known as Corridor disease, characterized by low levels of schizont parasitosis and piroplasm parasitaemia together with rapid mortality. By contrast, African buffalo, the long-established host of *T. parva*, and at least four other species of *Theileria* (Collins and Allsopp 1999; Norval et al. 1992; Bishop et al. 2004) remain asymptomatic following infection, although they typically harbour multiple *T. parva* genotypes.

A live infection and treatment (ITM) vaccination procedure results in long-term immunity to *T. parva* that is transmissible between cattle by ticks, especially to homologous parasite challenge. Research into immunological responses of cattle to ITM led to identification of CD8^+^ T cells as an important effector population conferring immunity (Mckeever et al. 1994). The use of CD8^+^ T cells from *T. parva*-immune cattle to screen cell lines transfected with parasite cDNA library pools subsequently led to the identification of specific genes encoding antigens that are targets of CD8^+^ T cell responses induced in cattle by ITM (Graham et al. 2006). The protein coding sequence of some of the CD8^+^ T cell target antigens is highly variable, but numerous studies have shown that much of the diversity in these antigen genes in *T. parva* is of buffalo origin. Based on the fact that *T. parva* has been introduced into cattle from buffalo relatively recently, it has been suggested that a majority if not all of the currently detected polymorphisms in CD8^+^ target antigens were probably generated in the buffalo, or possibly even the tick vector (Morrison et al. 2015). Although it is unclear whether they contribute to the *T. parva* resistance phenotype in buffalo, *T. parva*-specific CD8^+^ T cells capable of recognising both cattle-to-cattle transmissible and buffalo-derived *T. parva* have been isolated from African buffalo (Baldwin et al. 1988).

It has further been suggested that if the documented diversity of *T. parva* antigen genes arose as a result of immune selection in buffalo, it should be focused on those regions of the antigens that contain peptides capable of binding to buffalo class I MHC (Morrison et al. 2015). Such selection would be detectable with reference to cattle T cell responses only if bovine and buffalo class I MHC proteins shared peptide binding motifs. However, class I MHC haplotypes have yet to be analysed in buffalo and how similar the system is, in terms of allelic diversity and predicted peptide binding relative to that in cattle is unknown. On a practical level, overlaps in the peptide-binding repertoires of bovine and buffalo class I MHC proteins would have the implication that genes encoding antigens that are the target of CD8+ T cell responses in buffalo could be evaluated as recombinant vaccines in cattle.

In the present study, we have sequenced the peptide binding regions of the buffalo class I MHC transcripts. By the application of targeted high-throughput next-generation sequencing on both the Illumina and Roche 454 platforms, as well as Sanger sequencing of cloned full-length class I MHC transcripts, a remarkable molecular diversity in the buffalo class I MHC α1 and α2 domains has been revealed. Nucleotide substitution model discrimination statistics, distribution of codons with elevated posterior probabilities of positive selection, overlaps in predicted peptide binding specificities and transmembrane length variations provide novel insights into similarities and differences in the buffalo and cattle class I MHC systems.

## Materials and methods

### Samples

**Fig. 1:**
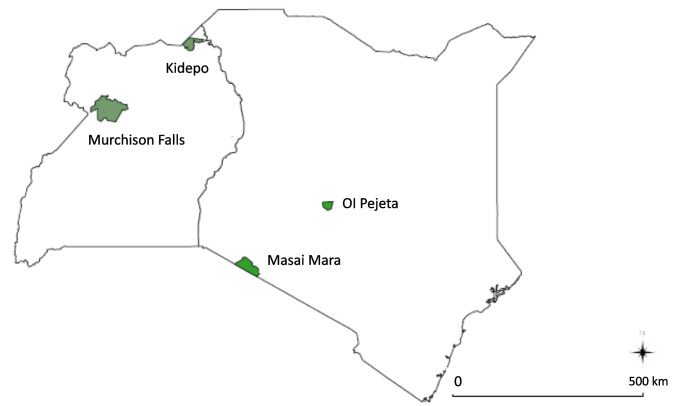
Map of Kenya and Uganda showing the location of buffalo sampling sites in Northern Uganda (Kidepo and Murchison Falls National Parks) and central Kenya (Ol Pejeta conservancy and the Maasai Mara game reserve)

A total of 47 African buffalo tissue samples (ear punches) were collected from national parks in Uganda and Kenya in RNAlater. From Uganda, samples were collected from the Murchison Falls (*n* = 22) and Kidepo Valley (*n* = 15) National Parks in Northern Uganda. Ten buffalo samples were collected from Ol Pejeta Conservancy in Kenya. Additionally, 20 archived buffalo samples, originally collected from Masai Mara, Southwest Kenya, were also included in the study. The buffalo sampled were immobilized by qualified veterinary personnel from the Uganda wildlife authority (UWA, research approval number COD/96/05) and the Kenya Wildlife Services (KWS, research approval number KWS/BRM/5001). The location of the four sampling sites is shown in Fig. 1.

### RNA extraction and oligo dT-primed cDNA synthesis

We isolated total cellular RNA from tissues that were stored in RNAlater and subsequently pulverized under liquid nitrogen, using TRIzol reagent according to the manufacturer’s instructions (Invitrogen). Total RNA was treated with DNase to remove contaminating genomic DNA. cDNA was synthesized from purified RNA using the Invitrogen SuperScript III reverse transcription system, with oligo(dT) primers.

### Sequence analysis of the peptide binding regions of buffalo class I MHC transcripts

- **Illumina Miseq next generation sequencing**
  We amplified cDNA of a 410 base pair polymorphic region of the class I MHC heavy chain from the 37 buffalo from the two Northern Uganda National parks and the ten animals from the Ol Pejeta conservancy in Kenya making 47 animals in total. The amplified region of the transcript spans exons 2 and 3 that encode residues that comprise the peptide binding groove. The PCR primers used, Bov7 (5′-GGCTACGTGGACGACACG-3′) and Bov 11 (5′- CCCTCCAGGTAGTTCCT-3′) are based on the most conserved sequences present in all publicly available cattle MHC-I sequence databases and are additionally conserved between cattle and the European Bison, which is classified within the same tribe as cattle, but in a different genus (Babik et al. 2012). The library preparation, sample indexing, and Illumina MiSeq run (300 bp paired-end strand-specific sequencing on Illumina MiSeq V3) were performed at LGC Genomics, Berlin (https://shop.lgcgenomics.com/).
- **Roche 454 Pyrosequencing**

A further group of 20 buffalo samples which had previously been sampled and archived, were also typed for class I MHC by Roche 454 pyrosequencing. These samples were originally collected from Masai Mara game reserve in Kenya. Frozen peripheral blood monocytes (PBMCs) were used for RNA extraction, and we then utilized the conserved primers (Bov7 and Bov11) described above, to amplify the 410 bp fragment spanning the peptide binding region from oligo dT-primed cDNA. To allow demultiplexing, each sample was uniquely tagged with a 10 base pair multiplex identifier (MID; Roche Diagnostics). Amplicons were pooled in equimolar amounts, and the manufacturer’s GS FLX protocol was used to perform emulsion PCR and GS FLX pyrosequencing at ILRI as previously described (Obara et al. 2016).

### Bioinformatic and statistical analysis of high throughput data

The quality control and artifact/chimera filtering steps were undertaken separately for the Illumina and the Roche 454 reads.

- **Initial quality control**
  The initial filters required that the following criteria were met: (a) a minimum read length size of 196 bp to allow reads to be assigned to either exon 2 or 3 (the read length was insufficient to allow overlap of exon 2 and 3-derived sequences, so these were analysed separately); (b) presence of complete barcodes and priming sequences. For the Illumina reads, this criterion was waived by allowing one or two mismatches or ambiguous bases (‘N’ calls) in the barcode when the barcode distances between all libraries on the lane allowed for it; (c) per base quality scores with at least a mean of Q30 (99.99% base call accuracy). For the Illumina reads, raw sequences after base calling were adapter clipped and demultiplexed using the Illumina bcl2fastq 2.17.1.14 software. For the 454 reads, the demultiplexing steps were accomplished using the flexible barcode and adapter removal tool (FLEXBAR; Dodt et al. 2012). We used the FASTX command line tools, FASTQ/A Trimmer and FASTQ Quality Filter for length trimming and quality filtering, respectively (http://hannonlab.cshl.edu/fastx_toolkit).
- **Disaggregation of ‘true’ class I variants from artifactual sequences**

A second set of algorithms was used to filter reads following a stepwise criterion to permit disaggregation of true alleles from artifactual sequences. Singletons were excluded by collapsing identical reads from each barcoded sample. Retained reads for each amplicon, ordered by frequency, were aligned using MAFFT (L-INS-i option) and the alignment stored in a structured query language (SQL) database. The SQL database was queried using dedicated Python scripts which implements a previously described iterative procedure to classify all collapsed reads as ‘putative artefact’ or ‘putative allele’ (Sommer et al. 2013). Reads that give an inconclusive result are termed ‘unclassified variants’. Chimeric sequences generated in amplicons were identified by code which tests whether a sequence read could be a combination of two different read clusters. This approach has previously provided accurate and repeatable genotype estimates of co-amplified class I MHC loci of African cattle (Obara et al. 2016). The quality of the assigned genotypes was assessed by comparing genotypes of individual animals obtained by amplicon-based NGS and Sanger sequencing.

### Sequence divergence between buffalo and African cattle class I MHC sequences

Our assessment of sequence divergence between buffalo and African cattle class I MHC was based on sequence matches using BLAST analysis. We also compared the patterns of nucleotide substitution between the buffalo class I MHC sequences and published African cattle class I MHC sequences. The African cattle sequences used for comparison included those derived from 17 Ugandan and Kenyan Ankole (Obara et al. 2016), 96 Cameroonian cattle, and 100 Kenyan Boran (Vasoya et al. 2016). For each alignment analysed, we used the Akaike information criterion (AIC) test, as implemented in jModelTest 2.1.10 (Posada 2008), to iterate through a set of evolutionary models that differ in ratios of nucleotide substitution and additionally allow for rate heterogeneity among sites and a proportion of invariant sites. We applied the AIC model discrimination statistics to an ensemble of 88 nucleotide substitution models and used Akaike weights to evaluate model fit. The models that gave superior fit to each data set were compared in terms of base frequencies, substitution rate parameters, and the proportion of invariant sites.

### A comparison of the distribution of codons with elevated posterior probabilities of positive selection at the buffalo and cattle class I MHC loci

We evaluated which of the alternative models of selective pressure is most consistent with buffalo and cattle MHC-I sequence data sets. We used CODEML from the PAML4 package (Yang 2007) to evaluate if there are positions in the buffalo and cattle MHC-I sequences encoding residues that show an excess of non-synonymous (*dN*) over synonymous substitutions (*dS*). *dN/dS(ω )*>1 is indicative of positive selection for amino acid substitutions. Models that were fitted to the data included: M1a – two discrete categories, one for purifying selection where ω<1, and the other for neutral selection where ω = 1 (Nielsen and Yang 1998; Yang et al. 2005); M2a – an extension of the M1a model, with an additional category for positive selection where *ω* >1 (Nielsen and Yang 1998; Yang et al. 2005); M7 – a continuous beta distribution of ω restricted to the interval (0;1), no positive selection allowed (Yang et al. 2000) and M8 – extension of M7 model, with additional, discrete category for positive selection (Yang et al. 2000). Akaike weights were used to evaluate model fit. If the best-fit model was M2a or M8, sites under positive selection were determined through the Bayes empirical Bayes (BEB) approach. We then ranked the codons by posterior probabilities of positive selection and compared the distribution of codons with elevated posterior probabilities of positive selection in the buffalo and the African cattle data sets.

For construction of the tree used as a basis for evaluating the models for positive selection, maximum-likelihood tree-search algorithms were implemented in PAUP 4.0 beta version using parameter estimates for the best-fit nucleotide model (Swofford 2002). We also calculated branch support using 1000 bootstrap replicates. For comparisons of tree topology and bootstrap values, we additionally performed maximum likelihood/rapid bootstrapping phylogenetic analysis using the Randomized Axelerated Maximum Likelihood (RaxML) tool available at the CIPRES portal (Stamatakis 2014) using the GTRGAMMA model and 1000 rapid bootstrapping replicates.

### Inference of functional overlaps between buffalo and cattle MHC -I molecules

Complete alpha 1 and alpha 2 domains are valuable for assigning class I MHC transcripts to functional clusters based on in silico predicted peptide binding specificities. For the purposes of indicating likely differences in peptide presentation between bovine and buffalo class I MHC and for analysis of haplotype configurations, we amplified full length class I MHC genes from oligo dT-primed cDNA templates from seven buffalo that had the largest number of most transcripts (putative alleles) based on analysis of the illumina data. Two of these buffalo were from KVNP, four from MFNP with a single animal from Ol Pejeta conservancy. We cloned PCR products from the seven buffalo into a plasmid vector, transformed bacteria, isolated 20 plasmid clones per animal, and purified these for bidirectional Sanger sequencing at LGC Berlin using M13 primers complementary to vector sequences flanking the insert. Standard chromatogram format (SCF) Sequence data was used to assemble consensus sequences in Geneious Prime 2019. Wherever present, ambiguous nucleotide sequence was re-examined in SCF files and adjusted appropriately. We analysed these full-length buffalo MHC-I transcripts for predicted peptide binding using the pan-specific machine learning neural network predictor NetMHCPan and compared their predicted peptide binding repertoires to bovine MHC-I alleles within the relevant MHC cluster.

## Results

### Analysis of class I MHC alleles transcribed in African buffalo based on Illumina reads

**Fig. 2:**
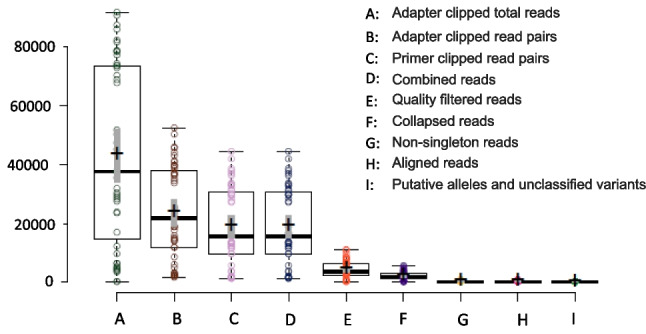
Box plot displaying the number of retained illumina reads after a stepwise filtering criterion. Central lines show the medians; box limits indicate the 25th and 75th percentiles as determined by R software; whiskers extend 1.5 times the interquartile range from the 25th and 75th percentiles; outliers are represented by dots; crosses represent sample means; bars indicate 95% confidence intervals of the means; data points are plotted as open circles. Thirty-seven Ugandan and 10 Kenyan buffalo samples were analysed

Multiplexed Illumina sequencing of the class I MHC antigen recognition site of 37 Ugandan (KVNP and MFNP) and 10 Kenyan buffalo (Ol Pejeta Reserve) generated 1,114,031 adapter clipped read pairs with a mean per-amplicon read pair count of 23703 (± 15647 SD; range = 1715 – 52333; Median = 21790). Figure 2 summarizes the results of the stepwise sequence filtering process that excluded reads categorized as of insufficient quality and also disaggregated putative alleles from artefactual sequences as described in Sommer et al. (2013).

Analysis of variants in the putative alleles retained following the stepwise filtering revealed high allelic diversity at the African buffalo class I MHC locus. Forty-two variants in exon 2 and 53 variants in exon 3 were identified among the cDNA samples from 47 buffalo that were sequenced using the Illumina platform. As shown in Table 1, all these allelic variants are present in at least 3 different buffalo, and some are present in more than 20 animals. As mentioned, the data is presented separately for exon 2 and exon 3 due to lack of overlap in the sequence reads. At the nucleotide level, these exon 2 variants had a mean nucleotide pairwise sequence similarity of 86.3% (± 5.3% SD; range = 99.4–74.4%), and the mean nucleotide pairwise sequence similarity for exon 3 alleles was 86.28% (± 5.0% SD; range = 99.4–74.4%). At the deduced amino acid level, the majority of nucleotide sequences translated into unique amino acid sequences, with the exception of seven exon 2 and four exon 3 variants that contained only synonymous substitutions. The mean frequency of these putative alleles within the population was 15.60% (± 10.1% SD; range = 42.55–6.38%). Analysis of sequence matches using BLAST revealed that these exon 2 and 3 alleles exhibited highest identity to all six classical class I MHC alleles present in cattle, but with differing frequencies (Table 1). Fourteen of the alleles appeared to be derived from non-classical Bola I loci (six encoded by exon 2 and eight encoded by exon 3).

**Table 1:** Sequence variation present within the second and third exons of class I MHC transcripts in two Ugandan and one Kenyan buffalo population

Exon 2				Exon 3			
Allele name	IPD BLAST hit		Allele frequency (no. of buffalo)	Allele name	IPD BLAST hit		Allele frequency (no. of buffalo)
	BoLA-I	% identity			BoLA-I	% identity	
E2_301	1*009:01	95.00%	8	E3_411	1*007:01	94.70%	4
E2_300	1*019:01	95.60%	7	E3_300	1*028:01	97.40%	7
E2_1185	1*074:01	94.40%	7	E3_305	1*075:01	93.80%	4
E2_660	1*009:01	94.40%	6	E3_301	1*042:01	97.20%	8
E2_305	1*009:01	93.90%	4	E3_666	1*061:01	93.40%	5
E2_223	1*042:01	96.10%	3				
E2_1182	1*031:01	91.70%	3				
				E3_2	2*026:04	94.40%	23
E2_101	2*030:01	97.20%	9	E3_102	2*026:04	94.90%	14
E2_658	2*046:01	91.10%	14	E3_406	2*076:01	94.70%	9
E2_297	2*005:01	91.60%	9	E3_1185	2*044:01	97.90%	7
E2_299	2*054:01	95.40%	6	E3_8	2*076:01	95.20%	6
E2_6	2*030:01	96.60%	6	E3_732	2*048:01	95.20%	6
E2_302	2*048:01	96.10%	5	E3_222	2*026:04	93.80%	4
E2_1667	2*030:01	95.50%	4	E3_33	2*026:04	94.90%	4
E2_224	2*012:01	95.00%	3	E3_185	2*048:01	88.50%	4
				E3_303	2*026:04	95.40%	3
E2_103	3*058:01	95.00%	6	E3_44	2*076:01	94.70%	3
E2_405	3*066:01	95.00%	13	E3_417	2*018:02	94.80%	3
E2_104	3*066:01	93.30%	11	E3_1398	2*076:01	96.80%	3
E2_10	3*066:01	92.70%	13	E3_1397	2*076:01	94.70%	3
E2_2	3*036:01	91.10%	23				
E2_8	3*035:01	91.50%	7	E3_4	3*059:01	96.30%	9
E2_4	3*010:01	94.90%	6	E3_3	3*068:01	94.20%	9
E2_102	3*036:01	91.10%	13	E3_143	3*036:01	93.50%	7
E2_406	3*052:01	92.20%	9	E3_10	3*036:01	94.10%	11
E2_732	3*035:01	95.00%	6	E3_6	3*059:01	96.30%	6
E2_390	3*066:02	95.40%	4	E3_103	3*035:01	96.30%	6
E2_882	3*036:01	93.90%	4	E3_299	3*036:01	95.70%	5
E2_417	3*066:02	93.90%	3	E3_302	3*036:01	94.80%	5
E2_1190	3*036:01	93.30%	3	E3_731	3*036:01	96.20%	5
E2_1398	3*066:02	94.40%	3	E3_390	3*010:01	95.70%	4
				E3_407	3*036:01	94.10%	4
E2_139	4*076:01	93.90%	6	E3_393	3*059:01	95.20%	4
E2_222	4*076:01	93.30%	4	E3_1667	3*068:01	96.30%	4
				E3_141	3*036:01	93.50%	3
E2_537	5*064:01	94.90%	3	E3_223	3*068:01	95.80%	3
				E3_537	3*010:01	94.10%	3
E2_411	6*034:01	96.60%	4	E3_752	3*059:01	95.80%	3
E2_1186	6*034:01	97.20%	4	E3_1182	3*036:01	94.40%	3
E2_44	6*034:01	95.00%	6	E3_391	3*036:01	93.00%	3
E2_733	NC3*001:01	94.40%	7	E3_658	4*024:02	92.80%	12
E2_7	NC4*001:01	97.20%	20				
E2_144	NC4*001:01	92.70%	13	E3_104	6*015:02	94.10%	10
E2_142	NC4*002:02	92.70%	15	E3_660	6*015:01	94.20%	6
E2_9	NC4*002:01	97.80%	8	E3_1592	6*015:02	95.20%	5
E2_298	NC4*001:01	97.80%	3	E3_1186	6*015:02	94.20%	4
				E3_224	6*015:01	95.80%	3
				E3_7	NC4*002:02	99.50%	20
				E3_142	NC4*002:02	95.40%	15
				E3_538	NC4*002:02	96.90%	13
				E3_144	NC4*003:01	90.80%	12
				E3_9	NC4*002:02	99.00%	7
				E3_733	NC4*002:02	94.90%	7
				E3_225	NC4*002:02	99.00%	6
				E3_5	NC4*003:01	90.30%	4

NC denotes non classical BoLA I alleles. The numbers preceding the asterisks (*) in the BoLA-I names denotes the classical/non-classical class I loci (1-6) to which the allele is assigned.

### African buffalo class I MHC data generated using Roche 454 pyrosequencing

A total of 104,666 quality sequences were identified from the 20 archived Masai Mara Kenyan buffalo samples generated using 454 pyrosequencing. Seventeen allelic variants were categorized as bona fide alleles in exon 2 (mean nucleotide pairwise sequence similarity of 89.7%, ± 3.5% SD) and 30 in exon 3 (mean nucleotide pairwise sequence similarity of 83.5%, ± 4.48% SD). All nucleotide sequences translated into unique amino acid sequences for exon 2, while two contained synonymous substitutions within the exon 3 group. The transcripts had similarities to eleven IPD BoLA-1 alleles, fifteen BoLA-2 alleles, twelve BoLA-3 alleles, a single BoLA-4 allele, and six BoLA-6 alleles. There were no alleles similar to BoLA-5 in the sampled group of animals.

Whereas there was extensive allele sharing within the populations, the Kenyan buffalo population in Maasai Mara (Table 2) contained exon 2 and 3 alleles that were largely distinct from those present in the two Ugandan buffalo populations (Table 1). Only two exon 2 variants were shared between these populations.

**Table 2:** Sequence variation present within the second and third exons of MHC class I genes in the Maasai Mara Kenyan buffalo population

Exon 2				Exon 3			
Allelename	IPD BLAST hit		Allele frequency (no. of buffalo)	Allele name	IPD BLAST hit		Allele frequency (no. of buffalo)
	BoLA-I	%Identity			BoLA-I	% Identity	
E2_31	1*009:01	93.90%	2	E3_253	1*021:01	94.80%	1
E2_320	1*042:01	96.90%	1	E3_455	1*042:01	96.20%	1
E2_471	1*023:01	92.80%	1	E3_482	1*009:02	95.30%	1
E2_206	1*009:01	96.40%	1	E3_27	1*019:01	94.80%	1
E2_80	1*074:01	92.80%	1	E3_59	1*061:01	95.30%	2
				E3_584	1*009:02	93.90%	1
E2_81	2*030:01	96.40%	1				
E2_353	2*005:01	95.90%	1	E3_7	2*047:01	100.00%	3
				E3_34	2*076:01	93.90%	13
E2_432	3*073:01	97.40%	1	E3_13	2*044:01	97.20%	4
E2_234	3*081:01	99.50%	1	E3_144	2*032:01N	99.10%	5
E2_134	3*066:02	94.90%	1	E3_4	2*060:02	96.30%	1
E2_5	3*011:01	96.90%	1	E3_224	2*076:01	94.40%	3
E2_1	3*081:01	100%	21	E3_3	2*060:02	96.70%	6
E2_4	3*081:01	99.50%	8	E3_204	2*016:03	97.20%	1
E2_207	3*066:02	93.80%	4	E3_404	2*022:01	94.80%	1
				E3_453	2*032:01N	98.60%	1
E2_43	4*076:01	93.90%	1	E3_21	2*026:04	94.80%	2
				E3_489	2*044:01	96.70%	1
E2_21	6*041:01	100.00%	4	E3_61	2*026:04	94.80%	1
E2_79	6*034:01	95.40%	5				
				E3_1	3*011:01	97.70%	2
				E3_8	3*059:01	94.40%	4
				E3_35	3*002:01	95.80%	4
				E3_457	3*050:01	96.20%	1
				E3_27	3*035:01	93.90%	1
				E3_145	6*013:02	94.80%	5
				E3_146	6*015:02	93.90%	1
				E3_252	6*014:02	97.20%	10
				E3_342	6*041:01	100.00%	1

NC denotes non classical BoLA I alleles. The numbers preceding the asterisks (*) in the BoLA-I names denotes the classical class I loci (1-6) to which the allele is assigned

**Table 3:**
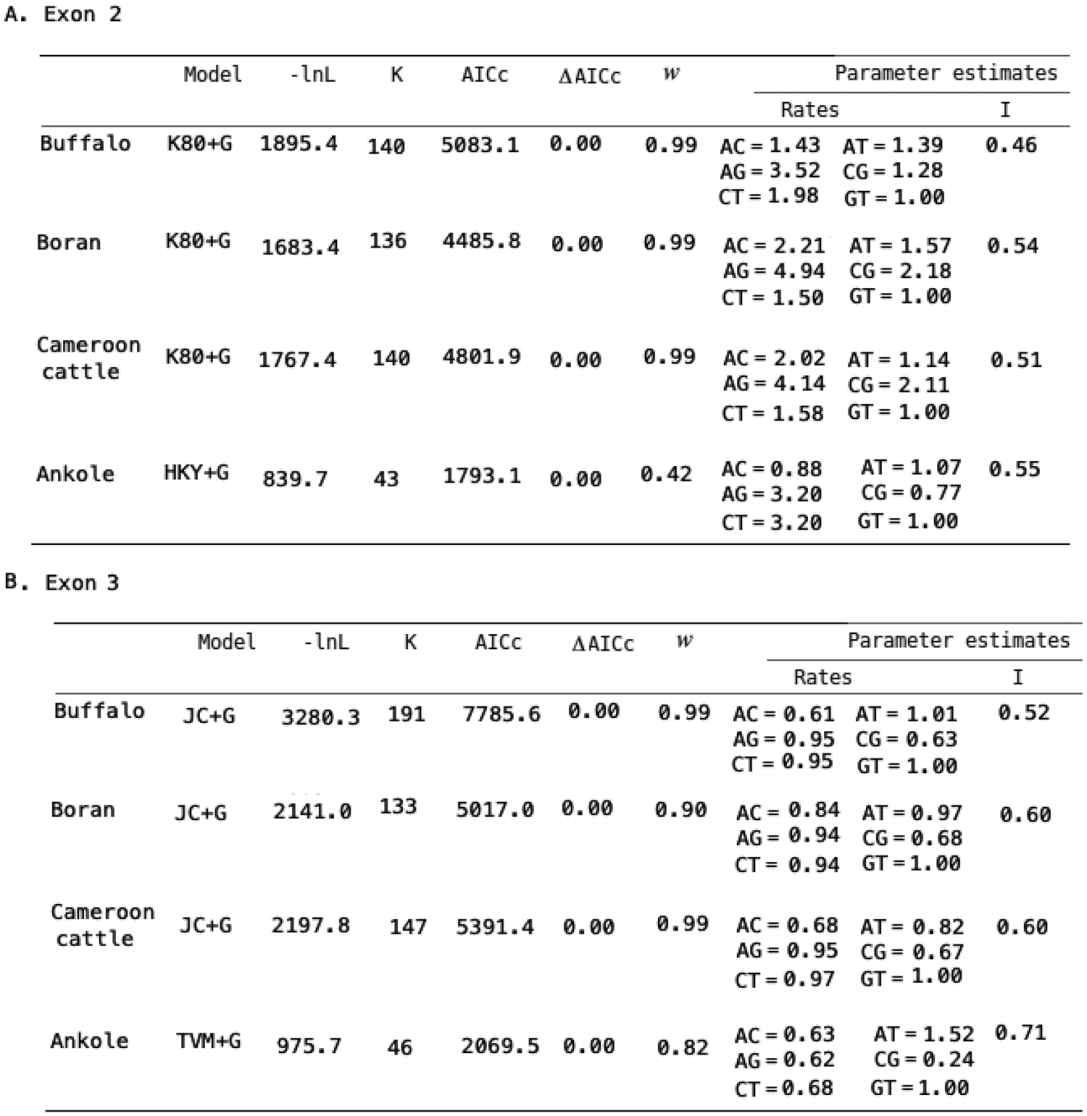
AIC ranking of a candidate set of nucleotide substitution models and parameter estimates for models with most support grouped by exon

-lnL and K refer to the negative log likelihood and the number of estimated parameters respectively. Model selection was based on identifying the model with the lowest Akaike Information Criterion corrected for small sample size (AICc). The probability that a model is the best of the candidate set given the data, was provided by the Akaike weights (w). Delta AICc denote the AICc difference between models. The model parameters estimated from the dataset include base frequencies (F), substitution rate parameters (Rates), and the proportion of invariant sites (I)

### Sequence divergence between buffalo and African cattle class I MHC sequences

**Fig. 3:**
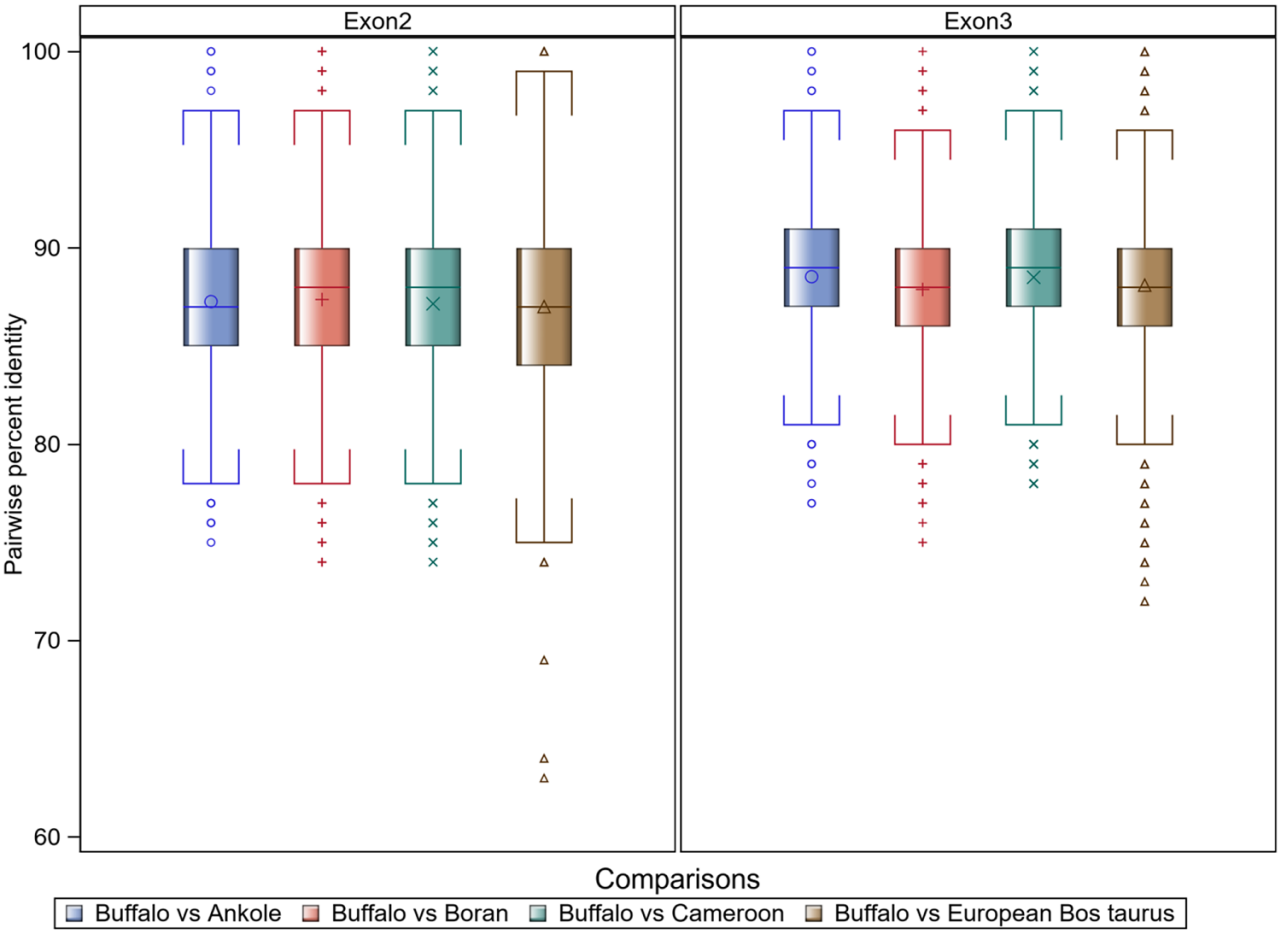
Boxplot showing the distribution of the percent pairwise sequence identities when the antigen recognition sites of class I MHC alleles of the African buffalo are compared to alleles from different African cattle breeds and alleles from European *Bos taurus*

As the closest bovid relative to the domesticated cow in Africa, and also the asymptomatic reservoir of multiple pathogens that cause disease in domestic cattle, the African buffalo is particularly interesting in terms of host-pathogen co-evolutionary mechanisms that potentially result in resistance. When the antigen recognition sites of class I MHC alleles of the African buffalo were compared to cattle (African *Bos Tauru*s and *Bos indicus*) alleles, the mean nucleotide percent pairwise sequence identity was as follows: buffalo vs Ankole cattle - exon 2 (87.27% , ± 3.96% SD; range = 100 – 75%), exon 3 (88.54 % , ± 3.42% SD; range = 100 – 77%); buffalo vs Boran cattle - exon 2 (87.38% , ± 4.05% SD; range = 100 – 74%), exon 3 (87.89% , ± 3.41% SD; range = 100 – 75%); buffalo vs Cameroon cattle - exon 2 (87.16 % , ± 4.30% SD; range = 100 – 74%), exon 3 (88.5% , ± 3.45% SD; range = 100 – 81%). The boxplot in Fig. 3 shows the distribution of the percent pairwise sequence identities when the class I MHC alleles of the African buffalo were compared to alleles from different African cattle breeds grouped in terms of exons. Sequences derived from the following European *Bos taurus* class I MHC haplotypes were also included in the analysis: A10, A11, A12 (w12B), A13, A14, A15, A15v, A19, A20 (v2), A31, BF1, H5 (New5), HP1.1, HP1.2, HP1.3, HP1.51.1, HP1.52.1, HP1.53.1, HP1.54.1, HP1.12.4, unHP1.74.1, unHP1.20.3 (Vasoya et al. 2016, 2021).

Even though the classical MHC molecules is the most polymorphic system currently known, the buffalo alleles were on average over 87% identical to the African cattle variants in the peptide binding region. What emerged from the AIC nucleotide substitution model discrimination statistics is a striking similarity in the evolutionary models with a substantially superior fit to the sets of aligned buffalo and cattle class I MHC nucleotide sequences. The Akaike weights (wi) strongly support models K80 + G and JC+G for the class I MHC exon 2 and 3 alleles, respectively, for buffalo as well as Boran and Cameroon cattle (Akaike weight = 0·99). Table 3 summarizes the likelihood scores, model selection criteria, and numerical values for the best fit model parameters. With the exception of Ankole cattle that had relatively fewer samples included in this analysis, buffalo and cattle class I MHC had the same best fit nucleotide substitution model, with identical support (Akaike weight = 0·99) and that in all the cases models that account Γ-distributed rates among sites were selected.

One relevant distinction between the buffalo and cattle class I MHC sequences that is evident in Table 2 relates to the proportion of invariant sites (I). The proportion of invariant sites is a description of heterogeneity among sites, and in this instance, it shows that although both cattle and buffalo MHC are extremely polymorphic, the polymorphism is more pronounced in the buffalo

### Full-length class I MHC sequence analysis

To complement the NGS data, we performed cloning and Sanger sequencing of full length class I MHC from seven buffalo. In total, 47 distinct full-length sequences were detected across these samples (GenBank OP852454-OP852499). The complete alpha 1 and alpha 2 domain sequences enabled us to explore clustering of predicted peptide binding specificities between buffalo and cattle alleles and to characterize sites under positive selection in the antigen recognition regions. We also used the 3’ portion of the full-length sequence data for a preliminary analysis of haplotype configurations and to investigate if loci are expressed in distinct combinations. The results of these analyses are presented below.

- **Sites of putative functional importance in the buffalo MHC coincides with positions known to be involved in peptide binding in cattle**

In testing if there are positions encoding residues that show an excess of non-synonymous over synonymous substitutions, maximum likelihood site-models (M1a, M2a, M7 and M8) were fitted to the full length buffalo class I MHC sequence data using CodeML as implemented in PAML v4.7 (Yang 2007). The Akaike weights (wi) strongly supported models allowing for a proportion of sites to evolve under positive selection relative to the gene evolution models tending towards neutrality - M2a over M1a and M8 over M7. Likelihood ratio tests of positive selection (Yang et al. 2005) implemented in EasyCodeML (Gao et al. 2019) similarly favored M2a over M1a and M8 over M7. Codons putatively under positive selection were determined by calculating probabilities under a Bayesian population genetics framework using the Bayes empirical Bayes (BEB) approach. Sites are likely to be under positive selection if their ω is > 1 with high probability (Nielsen and Yang, 1998).

**Fig. 4:**
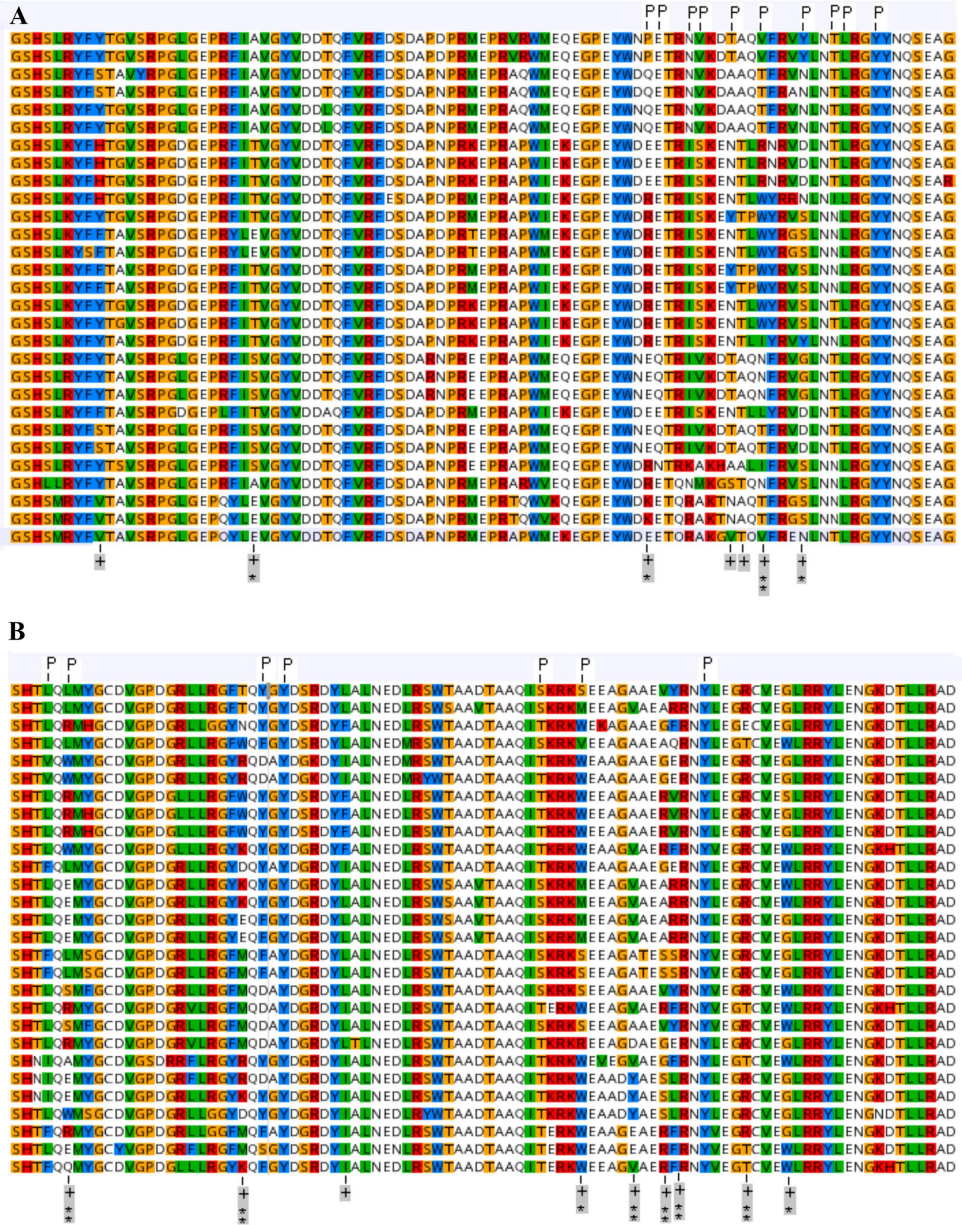
Amino acid alignment of buffalo class I MHC alleles. Plus sign ( +) marks sites under positive selection, codons which have ω > 1 with a significant Bayes Empirical Bayes probability are indicated by a single asterisk (*) if P > 95% or a double asterisk (**) if P > 99%. Codons known to be involved in antigen binding in cattle are marked with the letter P

This analysis revealed 16 sites in the peptide binding region of buffalo class I MHC genes with higher rates of non-synonymous to synonymous nucleotide substitutions than expected under neutral evolution. Twelve of these codons (4 in exon 2 and 8 in exon 3) have ω>1 with a significant Bayes Empirical Bayes probability (P>95%) and are therefore likely to be under positive selection. A single site in exon 2 and six sites in exon 3 has an even higher Empirical Bayes probability of ω>1 (P>99%). A proportion of the sites that exhibit an excess of non-synonymous over synonymous substitutions in buffalo class I loci are located in codons that are known to be involved in antigen binding in cattle. Another similarity with cattle is the observation that some residues under selection are seemingly not directly involved in peptide binding. Figure 4 shows a sequence alignment of buffalo class I MHC in which the distribution of residues under selection is identified relative to the peptide binding sites in cattle Fig. 4 (Panel A exon 2; Panel B exon 3).

b) **Broad clustering of peptide binding specificities between buffalo and cattle sequences**

**Fig. 5:**
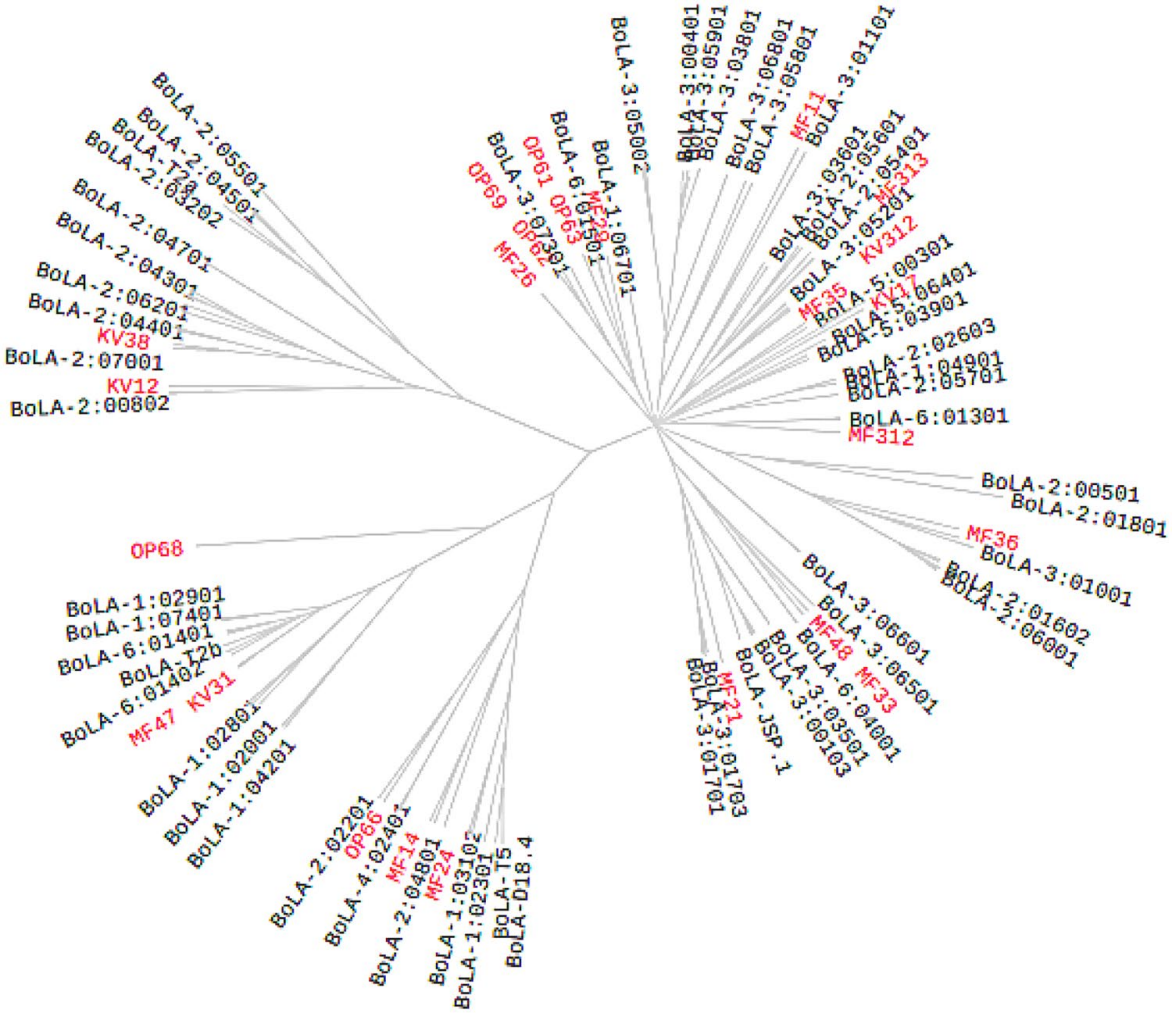
Class I MHC distance tree depicting the relationship between predicted peptide-binding specificities of bovine alleles (black branch labels) and buffalo alleles (red branch labels). Alleles with similar peptide binding specificities cluster together and the closer class I MHC alleles branch, the larger the overlap between their predicted peptide-binding repertoires. For clarity, where buffalo alleles cluster so closely together such that the allele names overlap, only one has been retained

Clustering of class I MHC alleles into groups with differing peptide binding potential at their antigen-binding sites enables comparative analysis of class I MHC functionality. For purposes of indicating likely similarities/differences in peptide binding profiles between buffalo and cattle, all the 47 full-length buffalo class MHC transcripts were analysed for predicted peptide binding using the machine learning neural network predictor NetMHCPan. For comparison, we also analysed predicted peptide binding for a set of full-length class I MHC transcripts derived from 102 reference BoLA-I alleles that are representative of the currently known class I functional diversity present in cattle (Pandya et al. 2015). The functional distance between any two alleles was derived from correlations between predicted peptide binding affinities and used as input for generation of the class I MHC distance tree. Branch support and consensus tree calculations were based on 100 bootstrap replicates. The MHCcluster output (Fig. 5) suggests broad clustering of peptide binding specificities between buffalo and cattle as the buffalo alleles (branches labelled in red) fall within cattle class I MHC supertypes (clusters of MHC alleles with similar binding specificity).

**Fig. 6:**
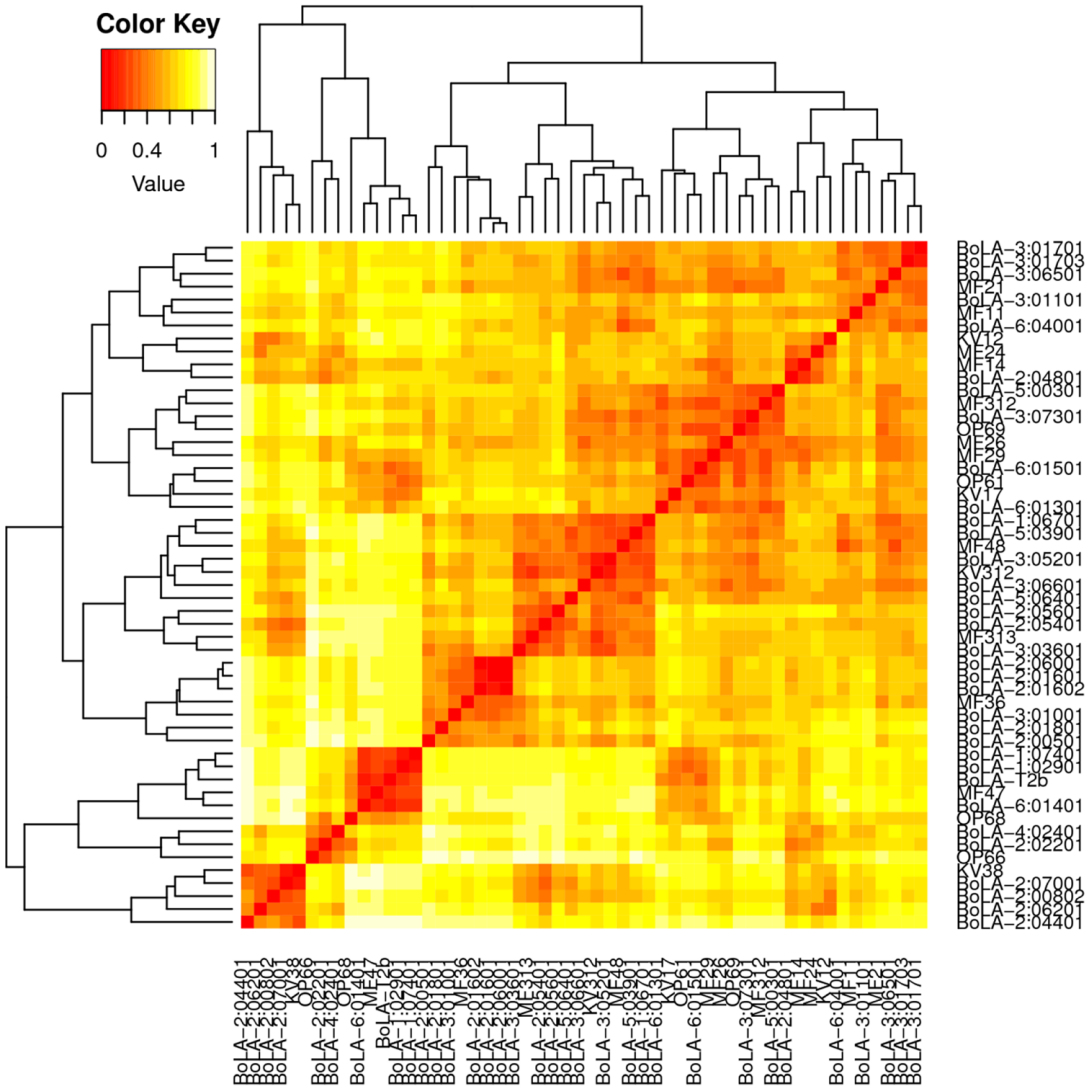
Heat-map visualization of the predicted peptide binding specificity overlaps between cattle and buffalo class I MHC transcripts inferred in MHCcluster. buffalo allele names are either prefixed ‘MF’, ‘KV’ or ‘OP’. All cattle alleles are prefixed ‘BoLA’. The colour key shows the MHC specificity distances between alleles. The bright orange represents the highest degree of overlap. Sequence logos describing predicted binding motif for the MHC molecule demonstrated overlap in key residues that underpin the binding specificity of bovine and buffalo class I MHC molecules. For clarity, where buffalo alleles cluster so closely together such that the allele names overlap, only one has been retained

The heat-map below in Fig. 6 adds to the illustration of the relatively low differentiation in the theoretical peptide binding spectrum between bovine and buffalo alleles.

For each MHC molecule, a position-specific scoring matrix (PSSM) was used as input for logo construction. This used information derived from the amino acid frequencies at each position of the 1% peptides that were predicted as binding most strongly to a specific molecule by NetMHCpan, using a set of 1,000,000 randomly chosen, 9mer peptides (Thomsen et al. 2013). The stack of residues, highlighted using the single letter code, at each position in the logo denotes the frequency of all 20 amino acids, and the height of these stacks represents the levels of amino acid conservation. The logo provides the following quantitative information: (i) the level of amino acid conservation at each position (the total height of the stack of letters), (ii) the relative frequency of a particular amino acid at that position (individual height of the amino acid symbol), and (iii) the under-representation of amino acids at each position (the negative region on the Y axis).

**Fig. 7:**
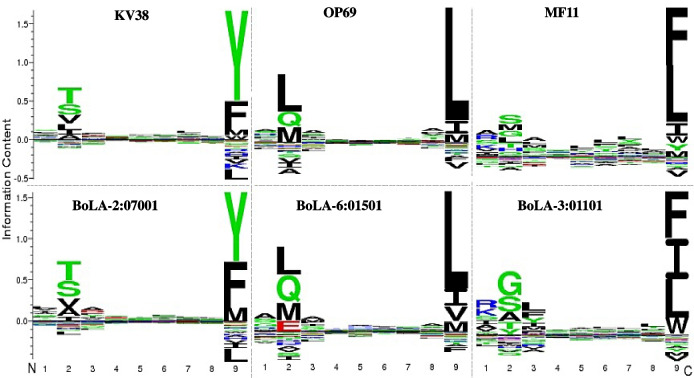
Logos illustrating predicted peptide-binding motifs for buffalo (upper panel) and bovine (lower panel) class I MHC proteins. The height of each stack of symbols (y-axis) represents information content (the level of amino acid conservation) in each position, the relative frequency of a particular amino acid at that position is represented by the individual height of the amino acid symbol and under-represented amino acids at each position are shown on the negative section of the y-axis

The sharing of peptide binding specificities between buffalo and cattle alleles infered from overlaps in the amino acid preferences at anchor positions is illustrated in Fig. 7. The predicted peptide binding analysis identified overlaps in the peptide-binding repertoires buffalo alleles KV3_8, OP6_9 and MF1_1 and the bovine alleles BoLA-2:07001, 6:01501 and 3:01101 respectively. These overlaps are not restricted to the peptide primary anchor residues (positions 2 and 9), but extend to several secondary anchors which are collectively responsible for high affinity binding. In the case of MF11 and BoLA-3:01101, the similarity between anchor residues at position 9 is much stronger than that of anchor residues at position 2.

### Configuration (haplotype) variation

**Fig. 8:**
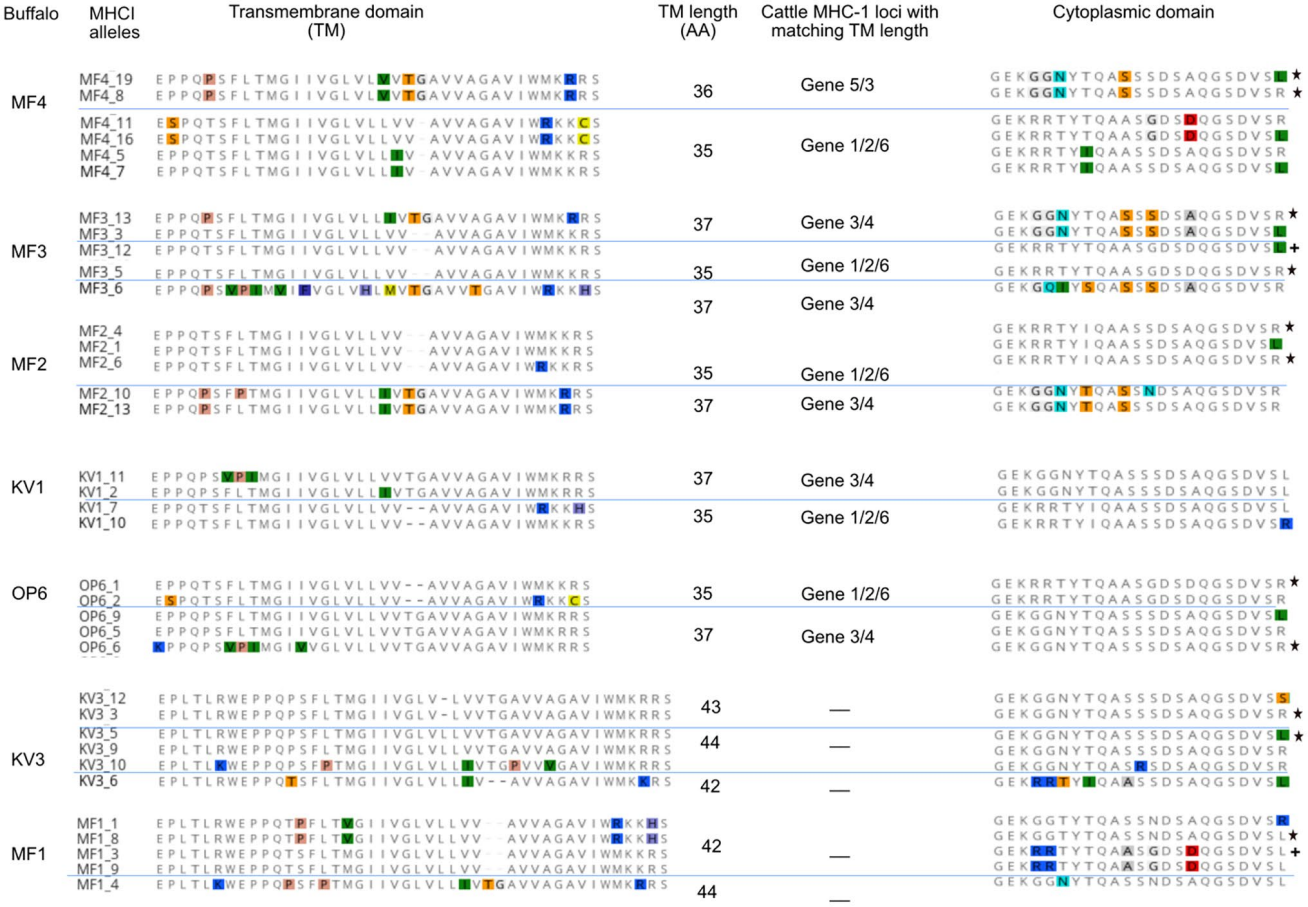
The predicted amino acid sequences for the transmembrane (TM) and cytoplsmic domains of buffalo class I MHC. The transmembrane lengths and the cattle loci with matching transmembrane lengths are also shown. Also shown are alleles identified per animal. Where two alleles from the same animal are identical in this region, an asterisk is used to denotes that only one is shown in the figure. Recurrence of alleles between individuals is indicated by the + sign

In contrast to the extracellular domains, the 3’ portion of class I MHC genes are known to show distinct locus-specific features (Birch et al. 2006). These include transmembrane length and locus-specific residues in the transmembrane and cytoplasmic domains. We used the 47 full-length class I MHC sequences described above to investigate transmembrane length variation and as well as amino acid polymorphisms in the transmembrane and cytoplasmic domains. As shown in Fig. 8, the full-length buffalo class I MHC sequences generated in this study had variable transmembrane lengths. The sequences of buffalo MF4 appear to fall into two groups - a 35 and a 36 amino-acid transmembrane region length. For buffalo MF3, class I MHC sequences with 35 and 37 amino-acid transmembrane regions can be distinguished. This is in common with buffaloes MF2, KV1 and OP6. The sequences derived from buffalo KV3 and MF1 had longer transmembrane lengths of either 42, 43, or 44 amino acids.

## Discussion

The African buffalo is the asymptomatic wildlife host of multiple species of livestock-infective apicomplexan protozoa in the genus *Theileria*, in addition to rickettsial pathogens such as the causal agent of contagious bovine pleuropneumonia (*Mycoplasma mycoides*) and heartwater (*Ehrlichia ruminantium*), together with multiple viruses. This makes the African buffalo interesting in terms of co-evolutionary mechanisms that result in resistance. A molecular arms race with pathogens is believed to drive the high allelic polymorphism and sequence divergence between alleles found in the classical class I MHC molecules. Here, we characterised the buffalo class I MHC exons that encode the section of the molecule involved in recognition of pathogen peptides leading to immune response to intracellular pathogens and compared the gene and predicted protein coding sequences to that of domestic cattle. The distribution of codons with elevated posterior probabilities of positive selection and the clustering of predicted peptide binding specificities were complementary in that they provided insight into the overlaps in the theoretical peptide binding spectrum between bovine and Buffalo alleles. In addition to extremely high allelic polymorphism in buffalo class I, transmembrane domain lengths reveal that the exact complement of expressed loci is variable between different animals.

The typical features of the MHC, specifically the extensive polymorphism and existence of multiple loci, constrain efficient and reliable genotyping. NGS techniques enable analysis of MHC diversity in large samples of animals, using pooled cDNA amplicons generated by co-amplification of multiple loci. We applied a deep sequencing strategy, based on a combination Illumina MiSeq technology and 454 pyrosequencing. However, a major problem is that not all reads revealed by these techniques represent legitimate sequence variants. In deconvoluting data generated from amplicon pools into that relevant at the individual level and disaggregating variants from artifacts and chimeras, we have made use of a stringent sequence filtering pipeline originally developed for MHC genotyping of non-model organisms (Sommer et al. 2013*).* As already mentioned, the data came from two different sets of animals: Northern Ugandan buffalo populations from Murchison Falls and Kidepo Valley (alleles identified in the Illumina sequencing) and a Kenyan buffalo population from Maasai Mara (alleles identified in the 454 sequencing). Principal component and admixture analyses based on whole genome sequences have demonstrated significant differences between the populations largely due to geographical barriers restricting buffalo migration. Population genetics studies, based thus far largely on a pan-Sub-Saharan Africa analysis of a mitochondrial DNA locus, have provided support for two subspecies: the East/Southern African buffalo and West/Central African buffalo. The buffalo populations in Northern Uganda from where we sampled have been shown to represent an overlap between the two subspecies (Smitz et al. 2013). Overall, analysis of the antigen recognition regions of buffalo class I, as well as the full-length sequences, revealed a remarkable molecular diversity in the buffalo studied.

The information on the allelic polymorphism and divergence of the bovine class I MHC loci has been derived from work on European *Bos taurus* breeds. Since it is becoming increasingly evident that different cattle breeds often carry distinct alleles, the recent in-depth class I data on Ugandan and Kenyan Ankole (Obara et al. 2016), Kenyan Boran, and Cameroonian cattle (Vasoya et al. 2016) represent an important addition to databases that are relatively sparse in terms of data on the MHC of African cattle. These breeds inhabit tropical or subtropical climates in East and Central Africa where the pathogen population is relatively similar to that encountered by the African buffalo, although their exposure to African pathogens is relatively recent (5000 as compared to approximately 5 million years). We used class I MHC data from these breeds as a basis for exploring shared features of Buffalo and cattle class I MHC.

We first computed a matrix of pairwise genetic distances (expressed as percentage difference) between every pair of buffalo and cattle MHC I sequences. This analysis demonstrated that buffalo and cattle class I MHC exhibit high sequence similarity in their alpha 1 and alpha 2 domains. Among the buffalo alleles, the pairwise nucleotide identities in exon 2 and exon 3 were 81.2–100% and 88.9–100%, respectively. When we compared the buffalo sequences to Ankole, Boran, and Cameroonian cattle alleles, the mean pairwise nucleotide identity was still 86%, indicating a similar degree of sequence divergence both within buffalo and between buffalo and cattle class I MHC. One of the highlights of the analysis was the extent of pairwise genetic dissimilarities of some of the European *Bos taurus* sequences to Buffalo class I alleles. One potential explanation is that whilst European *Bos taurus* has been subjected to artificial selection for production traits, the buffalo dataset is derived from ‘natural’ populations.

In examining the underlying shared features of buffalo and cattle MHC evolution, statistical models of the substitution process suggest that buffalo and cattle MHC are subjected to similar selective pressures and evolutionary constraints. Adequacy of the models was evaluated using the Akaike information criterion tests, and the same best-fit model of evolution was selected for both buffalo and cattle data sets (identical Akaike weights). However, the consistently lower proportion of invariable sites in buffalo class I MHC relative to cattle, despite the fact that there were more cattle samples than buffalo, suggests that buffalo might be capable of presenting a wider range of antigens to T lymphocytes. One interpretation of this result with respect to *T. parva* is that parasite diversity might be positively associated with MHC diversity in the two hosts. Buffalo are almost always infected with multiple genotypes of *T. parva* and other African protozoan and rickettsial pathogens and have co-evolved with these over a long timescale. In the case of *T. parva* infections, most of the variation in the CD8^+^ Tcell target antigens is found in isolates of buffalo origin. If CD8^+^ T cells are relevant to the *Theileria* tolerance phenotype in buffalo, this would require that the MHC alleles are highly divergent functionally in order to be able to bind polymorphic antigens from a diverse range of parasites.

In most species investigated so far, regions in MHC sequences coding for residues involved in binding antigens consistently exhibit signatures of positive selection, identified as an excess of non-synonymous mutations. We assessed whether sites of putative functional importance in buffalo class I MHC, inferred from dn/ds (ω) analysis, have any correspondence to known antigen binding sites in cattle. The phylogeny-based models of codon substitution assign sites into three classes: low ω, purifying selection, intermediate ω, nearly neutral and high ω, potentially positive selection and generated posterior probabilities for allocation in the positive selection class. This analysis revealed concordance in the distribution of codons with elevated posterior probabilities of positive selection in the buffalo class MHC and known antigen binding sites in cattle, suggesting sharing of some functional motifs between cattle and buffalo class I MHC.

MHC cluster output showed broad clustering of the buffalo and cattle alleles in the same supertypes (clusters of MHC alleles with similar physicochemical properties at their antigen-binding sites), which suggests low differentiation in their predicted peptide binding spectrum. This analysis uses the functional distance between any two alleles derived from correlations between NetMHCpan predicted peptide binding affinities as input for generation of a distance tree where alleles with overlaps in their peptide-binding repertoires cluster together. NetMHCpan is the artificial neural network-based T cell epitope prediction tool. Sequence logos showing the position specific peptide binding preferences in class I MHC peptide binding groove allowed closer scrutiny of overlaps in the peptide binding specificities of buffalo and cattle alleles. As shown in the logos for certain alleles (Fig. 4), the overlaps were not restricted to the peptide primary anchor residues (positions 2 and 9), but also extended to several secondary anchors (positions 1, 3, 6, and 7) which are collectively responsible for high affinity binding. This suggests that buffalo and cattle MHC could potentially select similar peptides with high affinity. Selection of high affinity peptides confers stability and immunogenicity to class I MHC and is one of the most important factors in establishing the specificity and intensity of a CD8+ T response.

The resulting MHC distance tree also highlights the fact that buffalo class I MHC alleles are functionally highly divergent (branches labelled in red in Fig. 2) since the alleles from the relatively few buffalo that were subjected to full-length class I MHC typing fall within all of the cattle supertypes. This suggests that in evolution, the large number of bovid pathogens in sub-Saharan Africa to which buffalo are exposed has selected for class I MHC alleles with a high functional capability, with the ability to bind to a more diverse array of peptides than cattle, which evolved primarily in West Asia.

It is important to note that use of pooled diversity measures generated by co-amplification of multiple loci constrains the ability to partition variation amongst loci in the absence of clear locus-specific characteristics. Humans are unusual in the sense that the same three expressed class I genes are present on all haplotypes and locus-specific characteristics within the coding region make assignment of sequences to loci straightforward. On the other hand, a degree of variation in the configuration of class I genes has been demonstrated in cattle (Ellis and Codner 2012), mice (Wroblewski et al. 1994), rats (Joly et al. 1996), horses (Ellis et al. 1995), pigs (Chardon et al. 1999), and some primates (de Groot et al. 2022; Cadavid et al. 1997). Buffalo class I MHC will need to be studied in sufficient detail to accurately define the number and nature of expressed MHC class I genes for a range of haplotypes. Since the class I MHC transmembrane length tends to be conserved within a locus, the six different transmembrane lengths in the buffalo sequences observed in this preliminary analysis suggest that each may represent a different locus. Furthermore, the observation that apparently based on transmembrane domain length and sequence motifs in exons 5–8 (Fig. 5), individual buffalo express alleles from a subset of the six putative loci, suggests that like cattle (Ellis and Codner 2012), African buffalo may exhibit configuration (haplotype) variation. If so, cattle are not unique in the subfamily in this aspect of their immunobiology, and this trait is a least 5 million years old, predating the divergence of the African buffalo and Auroch lineages. It will be of considerable interest in future to confirm whether this preliminary observation is correct, as well as to investigate other aspects of buffalo class I MHC in more depth. A further aim of future research should be the use of an assembly of partial sequences generated from two separate amplicons using primers that are universally conserved in buffalo sequences, or use of a combination of high-throughput short and long read transcript sequencing to enable pairing of exon 2 and 3 alleles.

## Data Availability

The African buffalo class I MHC sequence read data used in this study have been deposited to NCBI (GenBank OP852454-OP852499, OP960233-OP960302, OP960303-OP960398).
